# Identification of a Novel Immune Landscape Signature for Predicting Prognosis and Response of Endometrial Carcinoma to Immunotherapy and Chemotherapy

**DOI:** 10.3389/fcell.2021.671736

**Published:** 2021-07-23

**Authors:** Jinhui Liu, Yichun Wang, Jie Mei, Sipei Nie, Yan Zhang

**Affiliations:** ^1^Department of Gynecology, The First Affiliated Hospital of Nanjing Medical University, Nanjing, China; ^2^Department of Urology, The First Affiliated Hospital of Nanjing Medical University, Nanjing, China; ^3^Department of Gynecology and Obstetrics, Wuxi Maternal and Child Health Hospital, The Affiliated Hospital to Nanjing Medical University, Wuxi, China; ^4^Wuxi Clinical Medical College, Nanjing Medical University, Wuxi, China

**Keywords:** endometrial carcinoma, prognosis, tumor immune microenvironment, immunotherapy, chemotherapy

## Abstract

Uterine Corpus Endometrial Carcinoma (UCEC) is the most common gynecological cancer. Here, we have investigated the significance of immune-related genes in predicting the prognosis and response of UCEC patients to immunotherapy and chemotherapy. Based on the Cancer Genome Atlas (TCGA) database, the single-sample gene-set enrichment analysis (ssGSEA) scores was utilized to obtain enrichment of 29 immune signatures. Univariate, multivariate Cox regression and least absolute shrinkage and selection operator (LASSO) regression analyses were performed to generate an immune-related prognostic signature (IRPS). The biological functions of IRPS-associated genes were evaluated using GSEA, Tumor Immune Estimation Resource (TIMER) Database analysis, Mutation analysis, Immunophenoscore (IPS) analysis, Gene Expression Profiling Interactive Analysis (GEPIA), Genomics of Drug Sensitivity in Cancer (GDSC) and Immune Cell Abundance Identifier (ImmuCellAI). Potential small molecule drugs for UCEC were predicted using the connectivity map (Cmap). The mRNA and protein expression levels of IRPS-associated genes were tested via quantitative real-time PCR (qPCR) and immunohistology. Two immune-related genes (CCL13 and KLRC1) were identified to construct the IRPS. Both genes were related to the prognosis of UCEC patients (*P* < 0.05). The IRPS could distinguish patients with different prognosis and was closely associated with the infiltration of several types of immune cells. Our findings showed that patients with low IRPS benefited more from immunotherapy and developed stronger response to several chemotherapies, which was also confirmed by the results of ImmuCellAI. Finally, we identified three small molecular drugs that might improve the prognosis of patients with high IRPS. IRPS can be utilized to predict the prognosis of UCEC patients and provide valuable information about their therapeutic response to immunotherapy and chemotherapy.

## Introduction

Uterine Corpus Endometrial Carcinoma (UCEC) is the most common gynecological cancer. In 2018, 382,069 new cases and 89,929 deaths were reported worldwide (Bray et al., 2018). Despite the emergence of targeted therapy and immunological therapy, the incidence and mortality of UCEC have shown a consistent increase (Lortet-Tieulent et al., 2018). The overall 5-year survival rate can reach 75–86% (Gottwald et al., 2010), however, the survival time of patients with cancer metastasis or recurrence after treatment may drop below 16 weeks (Chaudhry and Asselin, 2009). Besides, the therapeutic regimens such as immunotherapy and chemotherapy are mainly designed according to the clinical stage of patients and regardless of the patients’ varying responses. Therefore, it is an urgent need of the scientific community to build a new prognostic model to identify patients that are at a high risk and suitable for certain regimens.

Surgery is the most preferred route to treat UCEC, commonly supported by radiotherapy and chemotherapy that are designed according to histopathologic parameters of the patients. Surprisingly, chemotherapy may exert different or even opposite effects on patients with identical pathological grade. Furthermore, there is limited evidence regarding the type of patients who can draw benefit from chemotherapy. To further complicate this, immunotherapy can trigger strong response in patients with DNA polymerase ϵ (POLE) mutation, microsatellite instability and high-tumor mutational burden (TMB), however, the difficulty of assessing these factors makes them unsuitable as prognostic markers.

Recently, the tumor immune microenvironment and infiltration of immune cells have been found to be associated with cancer development, prognosis and therapeutic response (Galon et al., 2013; Jain et al., 2016; Pages et al., 2018; Yu et al., 2018). Immune and stromal cells play critical roles in cancer biology. Immune related genes may regulate the infiltration of immune cells, a process that has close correlation with immunotherapeutic response (Binnewies et al., 2018). Therefore, we hypothesized that immune- related genes may be utilized to predict the prognosis and therapeutic response of UCEC patients. In this study, we identified two immune-related genes, their different expression levels have significant prognostic value, and developed a model for predicting the survival and therapeutic response of UCEC patients.

## Materials and Methods

### Data Sources and Clustering

Downloaded from the Cancer Genome Atlas (TCGA) database were data about mRNA expression data of 547 UCEC patients and clinical characteristics, including age, tumor grade, histological type and clinical stage from TCGA^1^ on Dec 1, 2019. All the mRNA expression data were derived from 552 tumor cases and 23 normal cases. 32 patients without well-annotated clinical information and survival time less than 30 days were excluded. After that, 515 patients were obtained. The tumor purity, infiltration level and stromal content were calculated through the ESTIMATE method (Yoshihara et al., 2013). The single-sample gene-set enrichment analysis (ssGSEA) scores were implemented via invoking the R package“GSEAbase”scores. to obtain the enrichment level of 29 immune signatures in each UCEC tissue by evaluating the mRNA expression level of UCEC patients and perform hierarchical clustering of UCEC using R package “hclust” (Barbie et al., 2009; Hanzelmann et al., 2013).

A total of 15 UCEC specimens and 15 adjacent tissues were obtained from patients at the Wuxi Maternal and Child Health Hospital, the Affiliated Hospital to Nanjing Medical University from 2018 to 2019 and routine written informed consent was obtained from all patients. These tissues were used to validate the mRNA and protein expression of KLRC1 and CCL13 in an external set.

### Differentially Expressed Genes and Immune-Related Genes

To identity the differentially expressed genes (DEGs) among all of the three groups, we first compared the DEGs between Immunity_L and Immunity_H, Immunity_L and Immunity_M, and Immunity_M and Immunity_H. After that, we imported the immune gene set from Immport database^2^. Then, the overlapping genes were obtained by Venn analysis.

### Gene Ontology and Kyoto Encyclopedia of Genes and Genomes Enrichment Analyses

We performed functional enrichment analyses to investigate the potential mechanisms of different hierarchical clustering based on 29 immune signatures. Gene ontology (GO) and Kyoto Encyclopedia of Genes and Genomes (KEGG) enrichment analyses were utilized to reveal the enriched biological process, cellular component, molecular function and signaling pathway. Terms with a false discovery rate (FDR) < 0.05 were listed using R package “ClusterProfiler”.

### Establishment of the Immune-Related Prognostic Signature

We divided all the cases into a training set and a testing set with the ratio of 1:1. We used the training set to identify the prognostic immune-related genes and to establish the IRPS. The testing set and entire set were used to validate its prognostic capability. First, a univariate Cox regression analysis was used to identify prognosis-related genes in the training set. The inclusion criterion was set at *P* < 0.05 and least absolute shrinkage and selection operator (LASSO) regression was utilized to minimize the overfitting. We then utilized multivariate Cox model to verify the correlation and developed an immune risk score model using the coefficients of multivariate Cox analysis. The risk score for patients in training set, testing set and total set was calculated using the following equation:

R⁢i⁢s⁢k⁢s⁢c⁢o⁢r⁢e=E⁢x⁢p⁢r⁢e⁢s⁢s⁢i⁢o⁢n⁢o⁢f⁢t⁢h⁢e⁢ 1s⁢t⁢g⁢e⁢n⁢e⋅c⁢o⁢e⁢f⁢f⁢i⁢c⁢i⁢e⁢n⁢t⁢+E⁢x⁢p⁢r⁢e⁢s⁢s⁢i⁢o⁢n⁢o⁢f⁢t⁢h⁢e⁢ 2n⁢d⁢g⁢e⁢n⁢e⋅c⁢o⁢e⁢f⁢f⁢i⁢c⁢i⁢e⁢n⁢t⁢+E⁢x⁢p⁢r⁢e⁢s⁢s⁢i⁢o⁢n⁢o⁢f⁢t⁢h⁢e⁢nt⁢h⁢g⁢e⁢n⁢e⋅c⁢o⁢e⁢f⁢f⁢i⁢c⁢i⁢e⁢n⁢t

Patients were then divided into high-risk and low-risk groups according the risk score.

### Validation of the IRPS

The receiver operating characteristic (ROC) curve was plotted to validate the prognostic value of IRPS. The area under the curve (AUC) was calculated using R package “survivalROC”. The survival analyses were conducted using Kaplan-Meier survival curves and “survival” R package. We also used the decision curve analysis (DCA) curve to obtain the predictive power of the IRPS and other clinical characteristics.

### Construction and Validation of a Predictive Nomogram

To fully expand the predictive power of a prognostic model, a nomogram was constructed based on the clinical characteristics of UCEC, including age, stage, grade and histological type. Validation of the nomogram was evaluated using calibration plot.

### Gene Set Enrichment Analysis

To identify potential biological mechanism related IRPS, we performed GSEA and GO analysis. KEGG terms with FDR ≤ 0.05 were considered enriched. Based on IRPS, patients were divided into different groups, the different expression genes with a fold change (FC) > 1 and an adjusted *P*-value < 0.05 were identified using R package “limma”. The GO analysis was then performed using the “clusterProfiler” R package.

### Estimate of Tumor-Infiltrating Immune Cells

We used the CIBERSORT tool to quantify 22 types of immunocyte fractions based on TCGA RNA-sequencing data. *P* < 0.05 was set as the threshold. *P* < 0.05 was set as the threshold.

### TIMER Database Analysis

TIMER is a comprehensive resource for systematical evaluations of the clinical impact of different immune cells on diverse cancer types^3^. We analyzed the expressions of KLRC1 and CCL13 in UCEC and evaluated their correlation with the infiltration of immune cells. Besides, correlations of KLRC1 and CCL13 expression with markers of several immune cells were also statistically evaluated using Spearman’s correlation and represented via scatterplots.

### TISIDB Database Analysis

The TISIDB online platform was used to analyze the correlation of KLRC1 and CCL13 expression with 28 immune infiltrating cells^4^.

### Mutation Analysis

We downloaded the mutation data of UCEC patients from the TCGA database^5^ and utilized the maftools to analyze the mutation data. The tumor mutational burden (TMB) score was calculated using following formula:

$$T\,M\,B=\frac{T\,o\,t\,a\,l\,m\,u\,t\,a\,t\,i\,o\,n}{T\,o\,t\,a\,l\,c\,o\,v\,e\,r\,e\,d\,b\,a\,s\,e\,d}\cdot {\left(10\right)}^{6}$$

### IPS Analysis

IPS can be generated in an unbiased manner using machine learning based on four major gene categories that determine immunogenicity. The IPS was calculated with z-scores of representative genes associated with immunogenicity. The IPSs of patients were obtained from the Cancer Immunome Atlas (TCIA)^6^.

### Immunotherapy Response Prediction

The response to immunotherapy was predicted using an online tool called Immune Cell Abundance Identifier (ImmuCellAI) (Miao et al., 2020), which can estimate the abundance of 24 immune cells from gene expression datasets, including RNA-Seq and microarray data, and predict the patient’s response to an existing immune checkpoint blockade therapy.

### Verification of Gene Correlation in GEPIA

To further verify the correlation of KLRC1 and CCL13 expression with immune cells markers, the Gene Expression Profiling Interactive Analysis (GEPIA)^7^ database was employed. Statistical analysis was performed using Spearman’s correlation.

### Chemotherapy Response and Candidate Small Molecule Drugs Prediction

The response of chemotherapy in UCEC patients was determined using a public database called Genomics of Drug Sensitivity in Cancer (GDSC^8^). The half-maximal inhibitory concentration (IC50) was estimated which represented the drug response. The potential small molecule drugs for UCEC were predicted using Connectivity map (CMap)^9^. This database comprises of genome-wide transcriptional expression data from small molecule drugs, and can discover the connections between drugs, genes and diseases through the variation of gene-expression profiles. These small molecule drugs were predicted based on 382 DEGs between high-risk and low-risk group with | log2fold change (FC) | > 1 and FDR < 0.05. The 3D structures of the three most significant drugs were obtained from Pubchem^10^.

### Quantitative Real-Time RT-PCR

Total RNA from 15 UCEC samples and 15 adjacent tissues was extracted using TRIzol reagent (Invitrogen) and the total RNA integrity were checked by RNA 6000 Nano kit. Before reverse transcription to cDNA, 4 × gDNA wiper Mix (Vazyme R323-01), DEPC and total RNA (1 μg) were resuspended and reacted at 42°C for 2 min to remove the residual genomic DNA from total RNA. PrimeScript^®^, RT reagent kit was used to synthesize the complementary RNA. The SYBR^®^, Premix Ex Taq^TM^ Kit (TaKaRa DRR041) was utilized to perform real-time quantification. The relative expression levels of target genes were normalized by GAPDH and estimated using the 2^–△^^△^^Ct^ method. The PCR primers are listed in Supplementary Table 2.

### Immunohistochemical Staining

The protein expression levels of CCL13 and KLRC1 were estimated via immunohistochemical (IHC) staining. Briefly, the tissues slides were deparaffinized, rehydrated and treated with 3% H_2_O_2_ for 15 min to eliminate endogenous peroxidase. Then, antigen retrieval was performed by heating the slides in sodium citrate buffer for 3 min. Next, the slides were incubated with rabbit anti-CCL13 or anti-KLRC1 primary antibodies (Affinity, Biosciences, 1:200) at 4°C overnight. The slides were washed and incubated with HRP-conjugated donkey anti-rabbit secondary antibodies (Abcam) for 15 min. The staining was visualized using DAB solution and samples were counterstained with hematoxylin.

Immunostaining of CCL13 and KLRC1 were analyzed by two pathologists who were blinded to the same information. The staining intensity score was defined on a scale of 0 to 3 in which 0 means no staining, 1 means mild staining, 2 means medium staining and 3 means intense staining. The percentage score of stained cells were also calculated on a scale of 1 to 4 in which 1 represents (0–25%), 2 = (26–50%), 3 = (51–75%) and 4 = (76–100%). In order to obtain the final score, the intensity score and percentage score were multiplied to reach the final score ranging from 0 to 12.

### Statistical Analysis

We adopted the R project (version 3.6.2; R Foundation) for all analysis^11^. The following R packages was adopted in this study (“pheatmap”, “rms” “ggplot2”, “forest plot”, “limma”, “glmnet”, “preprocessCore”, “GSVA”, “survminer”, “survival ROC”, “beeswarm”, “ggstatsplot”). Two-side statistical analyses were performed and samples with *P*-value < 0.05 were considered statistically significant.

## Results

### Construction of UCEC Subgrouping

**FIGURE 1:**
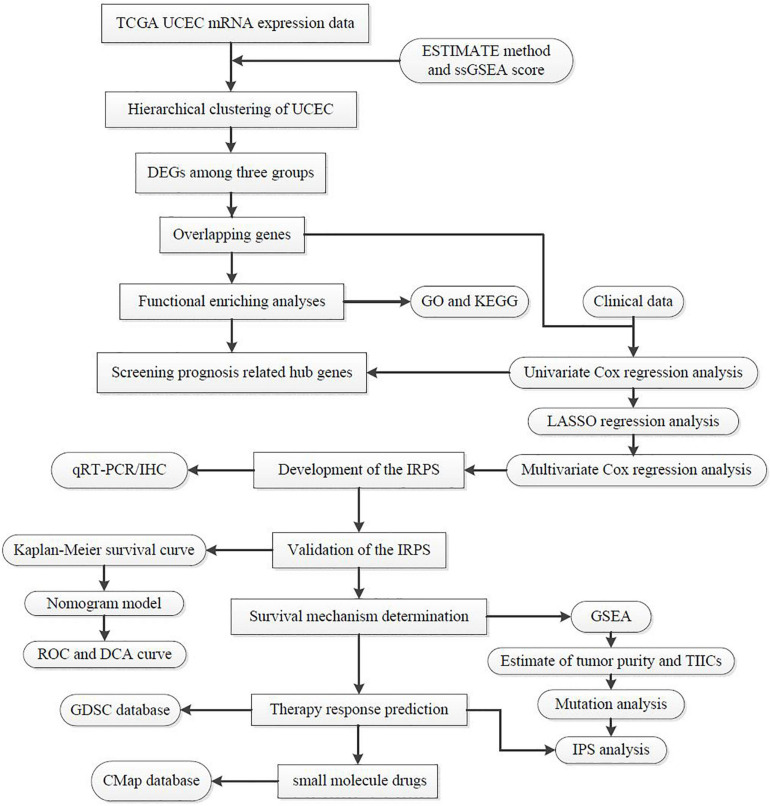
The workflow employed to identify small molecular drug targets for patients with high IRPS.

**FIGURE 2:**
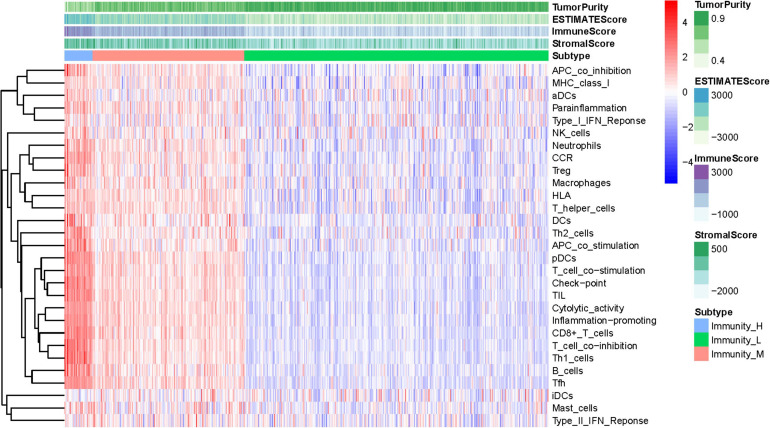
Hierarchical clustering of UCEC patients. Three distinct immune infiltration clusters, termed Immunity_H, Immunity_M and Immunity_L were defined with the help of ssGSEA scores of 29 immune signatures from TCGA database. The immune cells were highly expressed in the Immunity_H, and the low expressed in the Immunity_L group. Tumor purity, ESTIMATE Score, Immune Score and Stromal Score are shown in the above panel. Immunity_H (Immunity High), Immunity_M (Immunity Medium), and Immunity_L (Immunity Low) (The reference gene list can be obtained in the Supplementary Material A).

**FIGURE 3:**
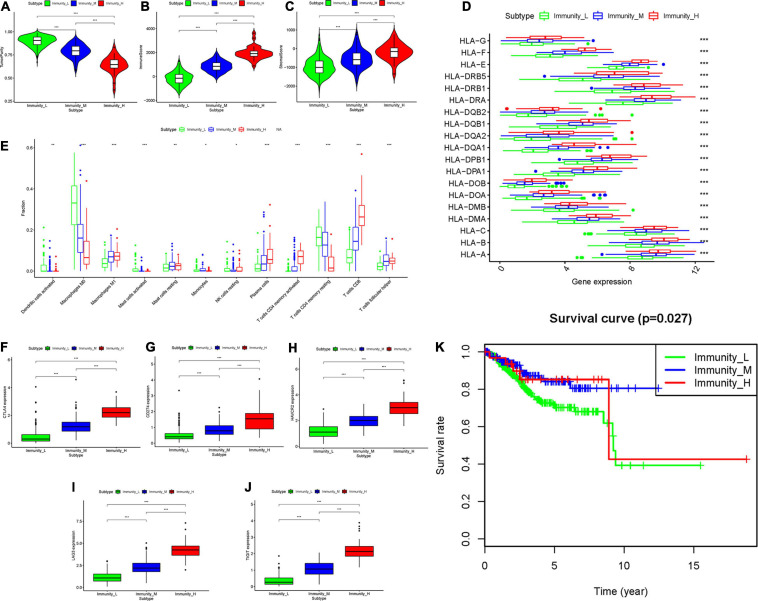
Three UCEC subtypes displayed different phenotypes. **(A–C)** The difference in Tumor Purity, Immune Score and Stromal Score in three UCEC subtypes. **(D)** The mRNA expression of HLA genes among UCEC subtypes. **(E)** The proportion of different immune cells in three UCEC subtypes. **(F–J)** The mRNA expression of 5 immune checkpoint molecules (CTLA4, CD274, HAVCR2, LAG3 and TIGHT) in three UCEC subtypes. **(K)** The survival curve exhibited that the prognosis of patients among UCEC subtypes is different. *0.01 ≤ *P* < 0.05; *0.001 ≤ *P* < 0.01; ***P < 0.001.

The total workflow is as shown in the following figure (Figure 1). With the help of the ssGSEA scores of 29 immune signatures and R package “hclust”, we divided the patients into three clusters according to immune infiltration: Immunity High (Immunity_H), Immunity Medium (Immunity_M), and Immunity Low (Immunity_L). The three distinct clusters, Immunity_H, Immunity_M, and Immunity_L, showed different immune activities. The hierarchical clustering map was shown in Supplementary Figure 1. We found that the patients in the Immunity_H group had higher ESTIMATE Score, Immune Score and Stromal Score and lower Tumor Purity (Figures 2, 3A-C) than other groups. Besides, the expression levels of most HLA genes were significantly higher in Immunity_H group than that in Immunity_L group (Figure 3D). The type of immune cells was different among three groups (Figure 3E). We also compared the expression of several immune regulators, including PD-1, PD-L1, TIM-3, LAG-3, and CTLA4. The expression levels of these immune regulators in Immunity_H group were all higher than those in Immunity_L group (Figures 3F–J). We then conducted Kaplan-Meier survival analysis which highlighted that patients in three groups had distinct clinical outcomes (*P* = 0.027, Figure 3K).

### Differentially Expressed Genes and Immune-Related Genes

To identity the differentially expressed genes (DEGs) among all of the three groups, we first compared the DEGs between Immunity_L and Immunity_H, Immunity_L and Immunity_M, and Immunity_M and Immunity_H and identified 2314, 411 and 1378 DEGs (Supplementary Figures 2A–C). Then, according to the gene set from Immport database (see text footnote 2), we obtained 1811 immune genes (Supplementary Material B). Then, the overlapping genes were obtained by Venn analysis. Finally, 89 overlapping genes were found differentially expressed in all four subgroups, which suggested to play a crucial role in immune status of UCEC (Supplementary Material C). Thus, the 89 overlapped DEGs were selected as key immune related DEGs for further analysis (Supplementary Figure 2D).

### Identification of Potential Biological Function-Related Genes

Go and KEGG analyses were performed which highlighted 89 biological function-related key genes in UCEC. We found that biological functions like such as T cell activation, leukocyte cell-cell adhesion, positive regulation of leukocyte activation, etc. were associated with the identified 89 genes. Furthermore, these genes participated in the KEGG pathways including Cytokine-cytokine receptor interaction, Natural killer cell mediated cytotoxicity, and Viral protein interaction with cytokine and cytokine receptor etc. (Supplementary Figures 3A–D).

### Development and Validation of the IRPS

**FIGURE 4:**
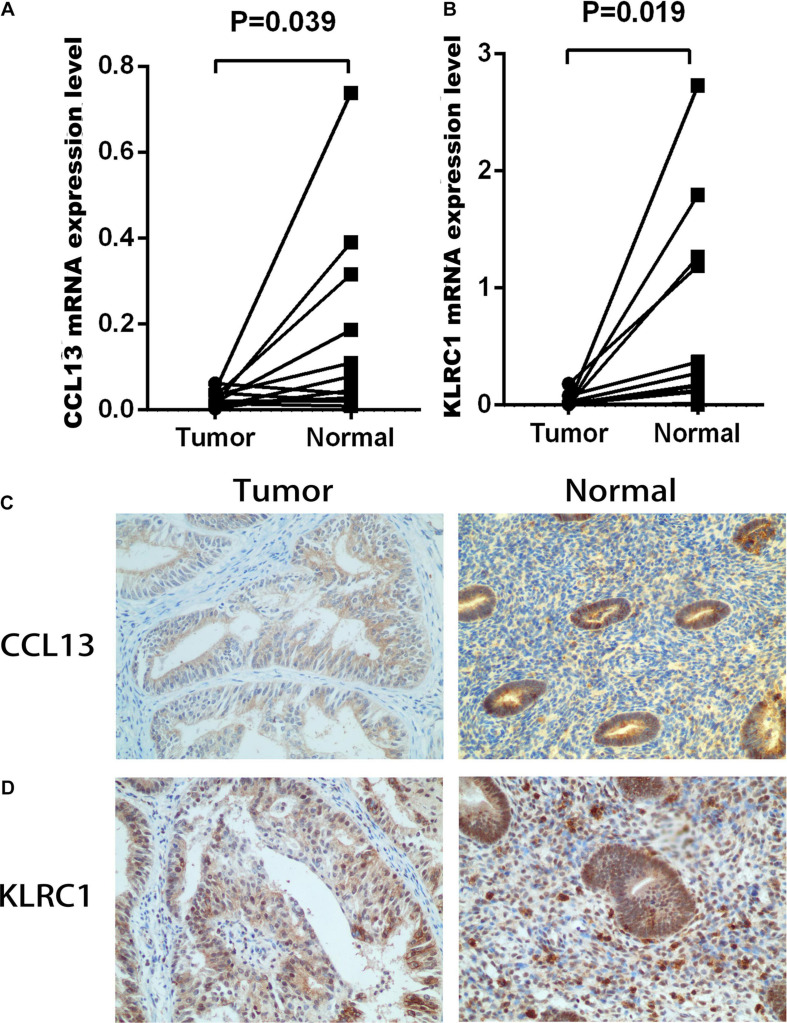
The mRNA and protein expression level of CCL13 and KLRC1 in UCEC tumor and the adjacent tissues. **(A,B)** The mRNA expression of CCL13 and KLRC1 in UCEC tumor and the adjacent tissues. **(C,D)** The protein expression of CCL13 and KLRC1 in UCEC tumor and the adjacent tissues observed using IHC.

To construct the IRPS based on 89 identified overlapping genes, the univariate Cox regression analysis was utilized to identify prognosis-related genes (Supplementary Table 1). 3 overlapping genes were identified. After that, we used LASSO Cox analysis to decrease overfitting (Supplementary Figures 4A,B). After analysis, three genes were all reserved, including KLRC1, CCL13 and LTA. All these genes were associated with the overall survival of UCEC patients (Supplementary Figure 5). We then performed multivariate Cox proportional hazards regression analysis to build the IRPS (Table 1). Two hub genes were reserved. The mRNA and protein expression of these two gens were presented in Figure 4. The mRNA expression of CCL13 and KLRC1 in tumor tissues were significantly lower than that in the normal tissues (Figures 4A,B). Similarly, the protein expression of CCL13 and KLRC1 was consistent with their mRNA expression (Figures 4C,D). The risk score was obtained according to the corresponding coefficients and the expression levels of hub genes. Risk score was calculated using the following formula:

**TABLE 1:** Multivariate Cox analyses based on 2 hub genes.

Gene	Coef	HR	95%CI	*P*-value
CCL13	−0.398	0.672	0.437–1.034	0.0709
KLRC1	−2.163	0.115	0.008–1.645	0.1111

R⁢i⁢s⁢k⁢f⁢a⁢c⁢t⁢o⁢r=[(-2.163)⋅K⁢L⁢R⁢C⁢1+(-0.398)⋅C⁢C⁢L⁢13]

A cutoff Risk score value of 1.40 was selected based on the median value of the risk score in the training set and used to divide the patients into low-risk (Risk score ≤ 1.4028) and high-risk (Risk score > 1.4028) groups.

**FIGURE 5:**
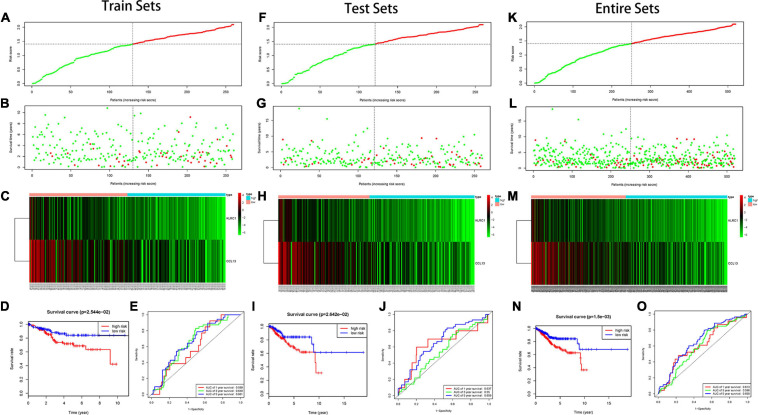
Construction of the IRPS. **(A–C)** The distribution of Risk score, survival status and expression of 2 hub genes in training set. **(D)** Kaplan-Meier survival curves of overall survival between high-risk and low-risk patients in training set. **(E)** 1-, 3-, and 5-year ROC curve of the predictive power of the IRPS in training set **(F–J)**. **(K–O)** display similar analyses which were conducted in the testing set and the entire set.

In the training set, we found the IRPS can distinguish risk score, survival status and expression of 2 hub genes as displayed in Figures 5A–C. Kaplan-Meier survival analysis showed statistical difference between two groups (Figure 5D), and the areas under the ROC curves (AUC) were 0.599, 0.649, and 0.661 for 1-, 3-, and 5-year survival, respectively (Figure 5E). The testing set cohort and the entire cohort were used to validate the prognostic power of the IRPS model. The distribution of risk score, survival status and expression of two hub genes in the testing and entire sets are presented in Figures 5F–H,K–M. Patients in high-risk group showed worse prognosis than low-risk group in both testing and entire sets (Figures 5I,N). ROC analysis revealed the prognostic accuracy of the IRPS in testing and entire sets (Figures 5J,O).

Furthermore, we analyzed the prognostic power of the IRPS with different clinical features in the entire set. We first represented the data in a heatmap to obtain the general distribution of risk score, hub genes and other clinical features (Supplementary Figure 5A) and then utilized subgroup Kaplan-Meier analysis to evaluate the prognostic value of IRPS in some specific conditions (Supplementary Figures 6B–G). We found the IRPS reached satisfactory prognostic discrimination in patients with age ≤ 60 (Supplementary Figure 6B), grade G1&G2 (Supplementary Figure 6C), grade G3&G4 (Supplementary Figure 6D), histological type endometrial (Supplementary Figure 6E), stage I&II (Supplementary Figure 6F) and stage III&IV (Supplementary Figure 6G).

### Construction and Validation of a Prediction Nomogram

We conducted the univariate and multivariate Cox regression analyses to determine whether the IRPS was an independent prognostic indicator for UCEC. According to univariate Cox regression analysis, the hazard ratio (HR) of risk score and 95% confidence interval (CI) were 2.717 (1.407–5.248), 1.783 (1.013–3.140), and 2.191 (1.425–3.370) in training, testing, and entire sets, respectively (Table 2). When turns to multivariate Cox regression analysis, the HR and 95% CI were 2.224 (1.137–4.350) and 1.949 (1.273–2.983) in training and entire set, respectively. However, in testing sets, the HR of risk score and 95% CI were 1.653 (0.956–2.859) (Table 2). According to the univariate and multivariate Cox regression analyses, the age, stage, histological type, grade and risk score are significant prognostic factors and should be involved in the construction of the prognostic models.

**TABLE 2:** Univariate and multivariate Cox regression analyses of the prognosis-related factors.

Variables	Univariate analysis			Multivariate analysis		
		
	HR	95%CI	*P*-value	HR	95%CI	*P*-value
**Training sets**						
Age	1.303	0.674–2.517	0.431	1.037	0.505–2.130	0.921
Stage	4.044	2.179–7.506	<0.001	2.902	1.496–5.629	0.002
Histological type	3.443	1.870–6.340	<0.001	1.597	0.729–3.500	0.242
Grade	2.869	1.327–6.202	0.007	1.881	0.774–4.568	0.163
RiskScore	2.717	1.407–5.248	0.003	2.224	1.137–4.350	0.020
**Testing sets**						
Age	2.465	1.253–4.851	0.009	2.346	1.159–4.751	0.018
Stage	4.126	2.306–7.383	<0.001	3.852	2.030–7.310	<0.001
Histological type	2.682	1.506–4.777	<0.001	0.918	0.462–1.822	0.806
Grade	3.996	1.864–8.565	<0.001	2.521	1.085–5.857	0.032
RiskScore	1.783	1.013–3.140	0.045	1.653	0.956–2.859	0.072
**Entire sets**						
Age	1.778	1.112–2.843	0.016	1.563	0.952–2.567	0.078
Stage	4.116	2.700–6.275	<0.001	3.400	2.152–5.371	<0.001
Histological type	3.044	2.003–4.624	<0.001	1.193	0.716–1.989	0.498
Grade	3.397	1.976–5.840	<0.001	2.137	1.163–3.928	0.014
RiskScore	2.191	1.425–3.370	<0.001	1.949	1.273–2.983	0.002

*HR, hazard ratio; CI, confidence interval.*

**FIGURE 6:**
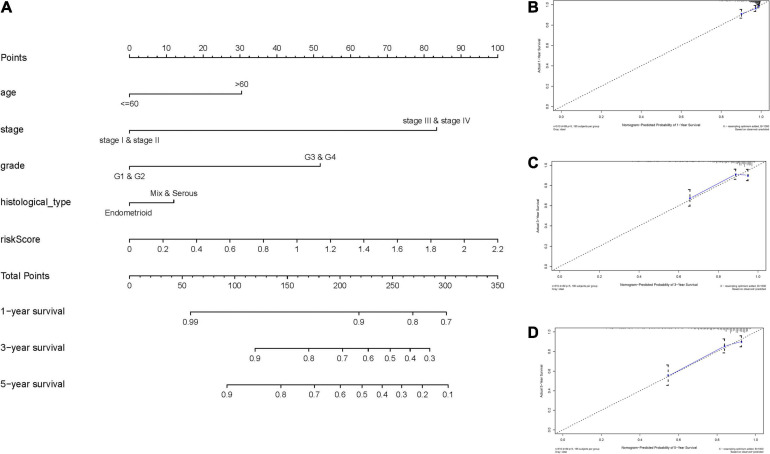
Construction and validation of a nomogram. **(A)** Nomogram to predict the probability of 1-, 3-, and 5-year OS of UCEC patients. **(B–D)** Calibration curves of the nomogram to predict the probability of OS at 1, 3, and 5 years.

**FIGURE 7:**
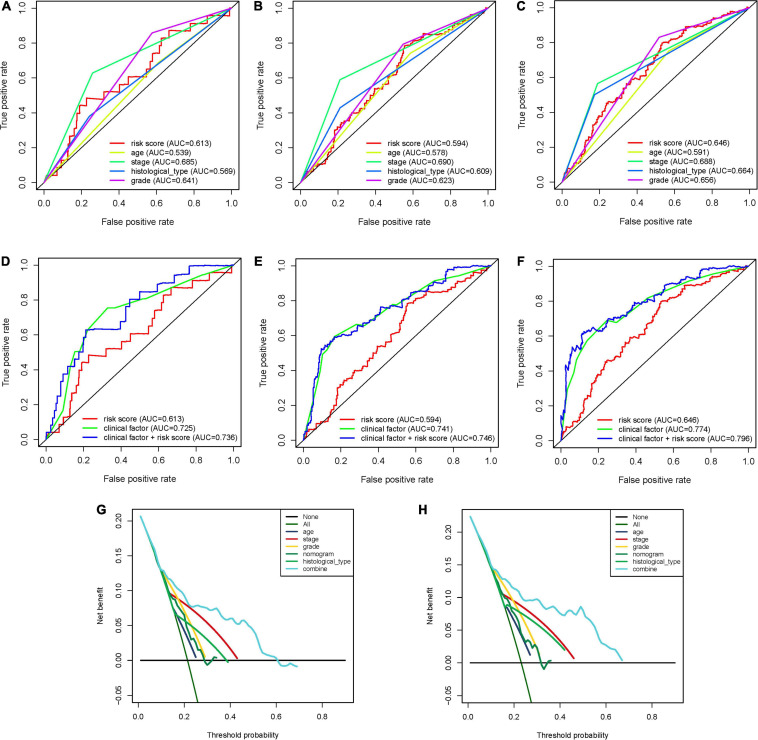
The predictive power of the IRPS and other clinical characteristics. **(A–C)** 1-, 3-, and 5-year ROC of IRPS and the other clinical characteristics. **(D,E)** 1-, 3-, and 5-year ROC of the combination of IRPS and the existing clinical factors. **(G,H)** The decision curve analysis (DCA) curves showed that the prognostic power of combining IRPS and clinical factors was superior to the existing prognostic factors.

To expand the prognostic power of the IRPS and other clinical characteristics, we constructed a nomogram that integrated age, clinical stage, grade, histological type, and risk score. Each parameter was assigned with a score and their total score was calculated. To validate the performance of the nomogram (Figure 6A), 1, 3, and 5-year calibration curves were constructed (Figures 6B–D), which revealed a close association between the predicted and actual curves. We further compared the AUC of IRPS and other clinical characteristics for 1-, 3-, and 5-year survival (Figures 7A–C) and found that the results were not suitable for clinical usage. However, when these factors were combined, the AUC reached 0.736, 0.746 and 0.796 for 1-, 3-, and 5-year survival, respectively (Figures 7D–F), suggesting the combination of IRPS and other clinical characteristics was highly reliable. This methodology was further confirmed by the decision curve analysis (DCA) (Figures 7G,H).

### Potential Biological Pathways Associated With IRPS

According to the GSEA analysis. We identified that pathways, such as axon guidance, basal cell carcinoma, glycosaminoglycan biosynthesis chondroitin sulfate, were enriched in the high-risk group, whereas autoimmune thyroid disease, B cell receptor signaling pathway, and chemokine signaling pathway were enriched in the low-risk group (Supplementary Figures 7A,B). Besides, we also identified several immune-related GO terms such as T cell activation, regulation of leukocyte activation, regulation of lymphocyte activation, regulation of T cell activation and leukocyte cell-cell adhesion (Supplementary Figures 6C,D).

### Correlation Between IRPS and Immune Cell Infiltration

**FIGURE 8:**
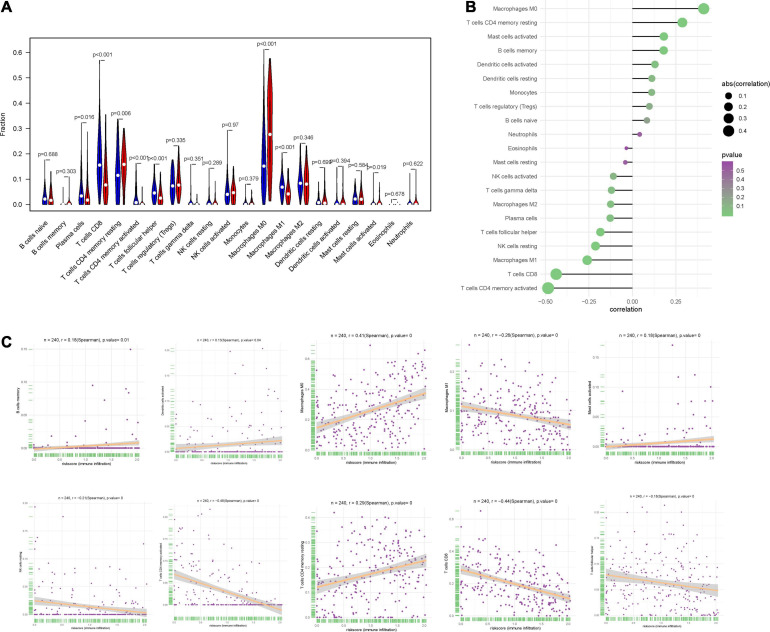
Correlation between IRPS and immune cell infiltration. **(A)** The landscape of immune cell infiltration in low-risk and high-risk groups. Th low-risk and high-risk groups are represented via blue and red violin, respectively. **(B)** The association between IRPS and immune cell infiltration. **(C)** The association between IRPS and each type of immune cell.

We used CIBERSORT to obtain the proportion of the 22 immune cells (Figure 8A) and found that the proportions of several types of immune cells, including plasma cells, CD8+ T cells, CD4+ memory T cell, follicular helper T cells and M1 macrophages, were higher in the low-risk group, but those of immune cells like, CD4+ memory T cells, M0 macrophages and mast cells were lower in the high-risk group. Besides, we also investigated the correlation between IRPS and different types of immune cells. The IRPS showed positive correlation with memory B cells, activated dendritic cells, M0 macrophages, mast cells, CD4+ memory T cells, and negative correlation with M1 macrophages, NK cells, CD4+ memory T cells, CD8+T cells and follicular helper T cell (Figures 8B,C).

### Correlation Analysis Between 2 Hub Genes and Immune Infiltration Level

To investigate the relationship between the two hub genes, tumor purity and the immune filtrating cells, we used the TIMER database to obtain their relationship. We first analyzed the correlations between CCL13 expression, tumor purity and immune infiltration level of 6 immune cells. The results showed that CCL13 expression level had negative correlation with tumor purity. Besides, CCL13 expression level had significant positive correlations with infiltrating levels of B cell, CD8+ T cell, CD4+ T cell, Macrophage, Neutrophil and Dendritic cell (Supplementary Figure 8A). Similarly, the KLRC1 expression level had negative correlation with tumor purity and positive correlation with infiltrating levels of B cell, CD8+ T cell, CD4+ T cell, Macrophage, Neutrophil and Dendritic cell (Supplementary Figure 8B). The above results were also validated using the TISIDB dataset (Supplementary Figures 9A–C). These results suggest that CCL13 and KLRC1 play a specific role in immune infiltration in UCEC.

We further revealed the relationship between the two hub genes and several immune markers, including CD8+ T cells, T cells (general), B cells, monocytes, TAMs, M1 and M2 macrophages, neutrophils, NK cells, DCs and several functional T cells. After adjustment by purity, the results showed that CCL13 expression level was associated with most immune marker sets of immune cells and different T cells except for the M1 Macrophage and Dendritic cell (Table 3). In addition, the KLRC expression level was associated with nearly all of the immune cells. These results were also verified using the GEPIA database (Table 4). In general, according to the above results, CCL13 and KLRC1 play a remarkable role in immune regulation in UCEC.

**TABLE 3:** Correlation analysis between two hub genes and immune cells markers in TIMER.

Description	Gene markers	CCL13				KLRC1			
			
		None		Purity		None		Purity	
					
		Cor	*P*	Cor	*P*	Cor	*P*	Cor	*P*
CD8+ T cell	CD8A	0.452	***	0.435	***	0.597	***	0.581	***
	CD8B	0.248	***	0.196	**	0.384	***	0.369	***
T cell (general)	CD3D	0.444	***	0.444	***	0.605	***	0.623	***
	CD3E	0.422	***	0.412	***	0.634	***	0.66	***
	CD2	0.468	***	0.466	***	0.624	***	0.624	***
B cell	CD19	0.157	**	0.119	0.041	0.385	***	0.399	***
	CD79A	0.307	***	0.296	***	0.454	***	0.436	***
Monocyte	CD86	0.343	***	0.301	***	0.542	***	0.543	***
	CD115 (CSF1R)	0.159	**	0.11	0.06	0.443	***	0.47	***
TAM	CCL2	0.303	***	0.272	***	0.282	***	0.253	***
	CD68	0.312	***	0.289	***	0.459	***	0.438	***
	IL10	0.136	*	0.125	0.032	0.236	***	0.199	**
M1 Macrophage	INOS (NOS2)	−0.019	0.659	−0.039	0.502	0.119	*	0.034	0.563
	IRF5	0.027	0.531	0.03	0.605	0.241	***	0.207	**
	COX2(PTGS2)	−0.015	0.732	−0.084	0.154	−0.033	0.446	−0.036	0.535
M2 Macrophage	CD163	0.376	***	0.324	***	0.393	***	0.365	***
	VSIG4	0.26	***	0.195	**	0.409	***	0.388	***
	MS4A4A	0.377	***	0.335	***	0.492	***	0.466	***
Neutrophils	CD66b (CEACAM8)	−0.092	0.031	−0.082	0.162	0.068	0.113	0.09	0.125
	CD11b (ITGAM)	0.228	***	0.158	*	0.441	***	0.466	***
	CCR7	0.33	***	0.344	***	0.482	***	0.518	***
Natural killer cell	KIR2DL1	0.164	**	0.091	0.12	0.445	***	0.423	***
	KIR2DL3	0.2	***	0.165	*	0.514	***	0.514	***
	KIR2DL4	0.29	***	0.322	***	0.693	***	0.696	***
	KIR3DL1	0.241	***	0.24	***	0.525	***	0.577	***
	KIR3DL2	0.193	***	0.21	**	0.485	***	0.493	***
	KIR3DL3	0.154	**	0.126	0.031	0.364	***	0.386	***
	KIR2DS4	0.182	***	0.136	0.02	0.465	***	0.512	***
Dendritic cell	HLA-DPB1	0.208	***	0.11	0.06	0.472	***	0.442	***
	HLA-DQB1	0.152	**	0.071	0.226	0.418	***	0.375	***
	HLA-DRA	0.182	***	0.084	0.152	0.475	***	0.435	***
	HLA-DPA1	0.234	***	0.143	0.014	0.539	***	0.519	***
	BDCA-1(CD1C)	0.027	0.529	0.01	0.868	0.345	***	0.344	***
	BDCA-4(NRP1)	0.081	0.059	0.075	0.203	0.304	***	0.238	***
	CD11c (ITGAX)	0.188	***	0.14	0.016	0.556	***	0.569	***
Th1	T-bet (TBX21)	0.45	***	0.411	***	0.631	***	0.617	***
	STAT4	0.282	***	0.24	***	0.493	***	0.46	***
	STAT1	0.27	***	0.248	***	0.296	***	0.244	***
	IFN-γ (IFNG)	0.495	***	0.489	***	0.498	***	0.481	***
	TNF-α (TNF)	−0.031	0.464	0.009	0.876	0.086	0.044	0.068	0.245
Th2	GATA3	0.195	***	0.164	*	0.237	***	0.19	*
	STAT6	−0.048	0.265	−0.103	0.079	0.065	0.129	−0.03	0.604
	STAT5A	0.119	*	0.053	0.367	0.274	***	0.303	***
	IL13	0.193	***	0.226	***	0.116	*	0.084	0.153
Tfh	BCL6	−0.085	0.046	−0.088	0.132	−0.005	0.902	0.054	0.356
	IL21	0.314	***	0.316	***	0.207	***	0.245	***
Th17	STAT3	0.02	0.643	−0.038	0.52	0.15	**	0.141	0.016
	IL17A	0.279	***	0.288	***	0.14	*	0.168	*
Treg	FOXP3	0.36	***	0.376	***	0.439	***	0.453	***
	CCR8	0.345	***	0.366	***	0.312	***	0.341	***
	STAT5B	0.065	0.129	0.044	0.449	0.107	0.013	0.111	0.058
	TGFβ (TGFB1)	0.103	0.016	0.114	0.052	0.223	***	0.213	**
T cell exhaustion	PD-1 (PDCD1)	0.421	***	0.371	***	0.466	***	0.458	***
	CTLA4	0.445	***	0.412	***	0.523	***	0.5	***
	LAG3	0.462	***	0.447	***	0.476	***	0.448	***
	TIM-3 (HAVCR2)	0.367	***	0.314	***	0.617	***	0.603	***
	GZMB	0.467	***	0.478	***	0.587	***	0.58	***

*Cor, R-value of Spearman’s correlation; None, correlation without adjustment. Purity, correlation adjusted by purity;* *P < *0.01;* **P < *0.001;* ***P < *0.0001.*

**TABLE 4:** Correlation analysis between two hub genes and immune cells markers in GEPIA.

Description	Gene markers	CCL13		KLRC1	
			
		*R*	*P*	*R*	*P*
CD8+ T cell	CD8A	0.59	***	0.65	***
	CD8B	0.37	***	0.52	***
T cell (general)	CD3D	0.47	***	0.59	***
	CD3E	0.49	***	0.65	***
	CD2	0.49	***	0.65	***
B cell	CD19	0.13	0.091	0.31	***
	CD79A	0.31	***	0.45	***
Monocyte	CD86	0.41	***	0.62	***
	CD115 (CSF1R)	0.29	**	0.52	***
TAM	CCL2	0.41	***	0.34	***
	CD68	0.42	***	0.52	***
	IL10	0.14	0.071	0.3	***
M2 Macrophage	CD163	0.39	***	0.48	***
	VSIG4	0.35	***	0.52	***
	MS4A4A	0.42	***	0.57	***
Neutrophils	CD66b (CEACAM8)	−0.13	0.093	0.022	0.77
	CD11b (ITGAM)	0.3	***	0.56	***
	CCR7	0.38	***	0.51	***
Natural killer cell	KIR2DL1	0.12	0.12	0.44	***
	KIR2DL3	0.3	***	0.54	***
	KIR2DL4	0.36	***	0.75	***
	KIR3DL1	0.35	***	0.5	***
	KIR3DL2	0.21	**	0.56	***
	KIR3DL3	0.25	***	0.39	***
	KIR2DS4	0.2	**	0.41	***
Dendritic cell	HLA-DPB1	0.3	***	0.56	***
	HLA-DQB1	0.22	**	0.36	***
	HLA-DRA	0.28	***	0.53	***
	HLA-DPA1	0.36	***	0.6	***
	BDCA-1(CD1C)	0.12	0.13	0.35	***
	BDCA-4(NRP1)	0.14	0.057	0.36	***
	CD11c (ITGAX)	0.26	***	0.59	***
Th1	T-bet (TBX21)	0.48	***	0.67	***
	STAT4	0.37	***	0.48	***
	STAT1	0.24	*	0.37	***
	IFN-γ (IFNG)	0.56	***	0.63	***
	TNF-α (TNF)	−0.022	0.77	0.041	0.6
T cell exhaustion	PD-1 (PDCD1)	0.45	***	0.55	***
	CTLA4	0.52	***	0.6	***
	LAG3	0.42	***	0.52	***
	TIM-3 (HAVCR2)	0.4	***	0.66	***
	GZMB	0.48	***	0.66	***

*P < *0.01;* **P < *0.001;* ***P < *0.0001*.

In addition, we analyzed the relationships of the mutants of these 2 hub genes with immune infiltrates in UCEC. Compared with the immune infiltration levels in samples with wild type signatures, diverse forms of mutation in two hub genes could inhibit the immune infiltration levels of several immune cells, including CD8+ T cell, macrophages and dendritic cells (Supplementary Figures 8C,D).

### Correlation Between IRPS and Mutation Profile

**FIGURE 9:**
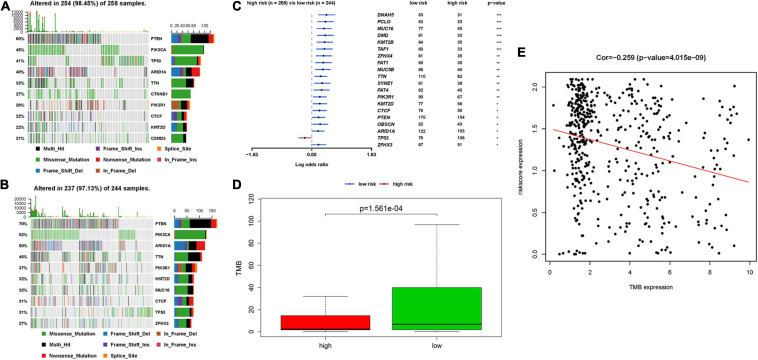
The mutation profile and TMB among high-risk and low-risk group. **(A,B)** The top 10 mutated genes in high-risk and low-risk group. **(C)** The most frequently mutated genes in high-risk and low-risk group. **(D)** The TMB in high-risk and low-risk group. **(E)** The relationship between TMB and IRPS. TMB: tumor mutational burden.

The relationship between IRPS and mutation profile was evaluated in UCEC patients using somatic mutation data. The top 10 mutated genes in high-risk and low-risk group are shown in the Figures 9A,B. And the most frequently mutated genes in high-risk and low-risk group are presented in Figure 9C. The results revealed that somatic mutation was more frequently observed in the low-risk group. And the TMB scores in low-risk group were significantly higher than that in high-risk group (*P* < 0.05, Figure 9D). Further results revealed that TMB score had negative correlation with IROS (*P* = 4.015e-09, Figure 9E).

### Correlation Between IRPS and Two Therapeutic Regimens

**FIGURE 10:**
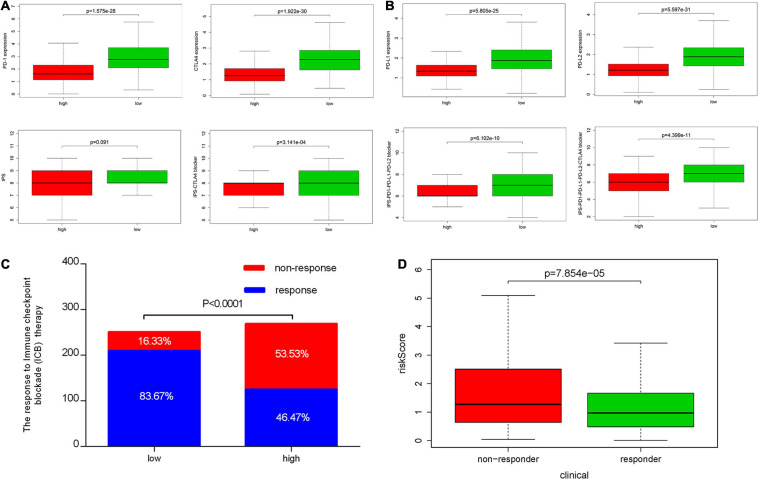
Correlation between IRPS and immune checkpoint molecules and the predicted response to immunotherapy. **(A)** The gene expression profile of PD1, CTLA-4, PD-L1 and PD-L2 in low-risk and high-risk group. **(B)** The association between IPS and the IRPS in UCEC patients. **(C)** The different immunotherapy response rates in low-risk and high-risk group. **(D)** The relationship between IRPS and immunotherapy response.

We also analyzed the expression of four immune checkpoint molecules in high-risk and low-risk groups. The results revealed that IRPS was negatively corelated with the listed four immune checkpoint molecules (Figure 10A). Besides, we performed IPS analysis to acquire immunogenicity. The results showed that four molecules displayed higher scores in the low-risk group (Figure 10B). Besides, according to the ImmuCellAI, patients in the low-risk group showed higher immunotherapy response rate compared with patients in the high-risk group (Figures 10C,D), which implied that patients in the low-risk group would benefit from immunotherapy.

**FIGURE 11:**
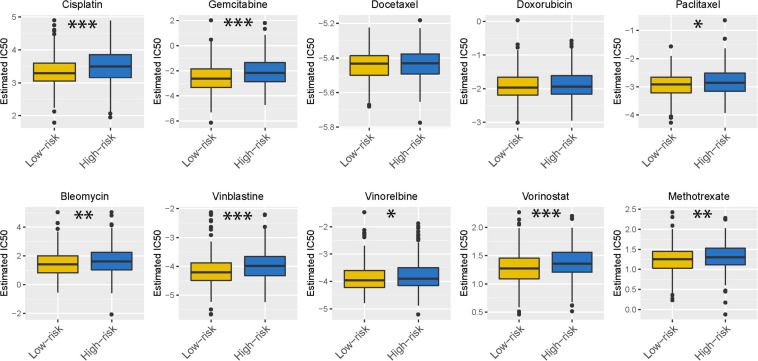
Chemotherapeutic response in the high-risk and low-risk groups. *0.01 ≤ *P* < 0.05; **0.001 ≤ *P* < 0.01; ***P < 0.001.

Chemotherapy is the most common way to treat UCEC cancer, in this research, we used GDSC database to predict the likelihood of response to several common chemotherapy drugs. We estimated the IC50 of each sample and observed a significant difference of IC50 between high-risk and low-risk groups among eight chemo drugs. Patients in the low-risk group were more sensitive to commonly administered chemodrugs (*P* = 1.467e-05 for cisplatin, *P* = 4.412e-06 for gemcitabine, *P* = 0.039 for paclitaxel, *P* = 0.002 for bleomycin, *P* = 1.458e-06 for vinblastine, *P* = 0.048 for vinorelbine, *P* = 4.620e-05 for vorinostat, *P* = 0.005 for methotrexate) (Figure 11). In contrast, the chemotherapeutic response of Docetaxel and Doxorubicin was not significantly different between both groups.

### Potential Small Molecular Drugs for UCEC

**FIGURE 12:**
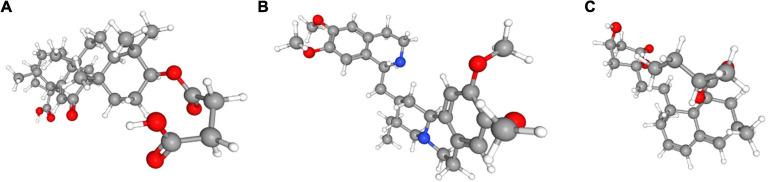
The 3D structure of the three small molecule drugs for UCEC. **(A)** carbenoxolone, **(B)** emetine, and **(C)** lovastatin.

In order to explore new therapeutic regimens for UCEC, the Cmap database was employed (Lamb et al., 2006). We found eight associated small molecule drugs that are listed in the Table 5. Among these small molecule drugs, the 3D chemical structures of three most significant small molecule drugs were obtained from PubChem (Figure 12).

**TABLE 5:** Results of CMap analysis.

Cmap name	Mean	*n*	Enrichment	*p*	Specificity	Percent non-null
Carbenoxolone	−0.332	4	−0.807	0.00271	0	50
Emetine	−0.6	4	−0.713	0.01369	0.1941	75
Lovastatin	−0.367	4	−0.704	0.01578	0.0233	50
MG-262	−0.567	3	−0.794	0.01787	0.1417	66
Piperlongumine	0.368	2	0.897	0.02181	0.1234	50
Megestrol	−0.406	4	−0.677	0.02425	0.0068	50
Semustine	−0.398	4	−0.653	0.03364	0.1111	50
Trimethoprim	−0.442	5	−0.577	0.04055	0.0449	60

## Discussion

UCEC is the most common tumor affecting female reproductive system, with a 5-year survival rate of 16% in patients with distant metastasis (Siegel et al., 2019). To date, the therapeutic regimens, such as immunological therapy and chemotherapy, are mainly designed according to the clinical stages of the tumor. Due to physiological differences, not all the patients can benefit from the current therapeutic regimens (Brooks et al., 2019). To overcome this challenge, in this research, we developed a model for predicting the survival and therapeutic response of UCEC patients using two immune-related genes.

We first estimated the relative levels of 24 immune cells based on the training set online data and used hierarchical clustering analysis to profile the infiltration of immune cells. The results revealed that the infiltration of immune cells varied much among UCEC patients. We found different tumor purities, immune scores, stromal scores, fractions of different immune cells, expression of several HLA genes and expression of five immune checkpoint molecules (CTLA4, CD274, HAVCR2, LAG3 and TIGHT) among UCEC subtypes. These results strongly suggest that tumor immune microenvironment has different landscapes in UECE patients. Emerging evidence demonstrates that tumor immune microenvironment is closely associated with the prognosis of several cancers^13^ (Liu et al., 2020). In this research, we found the overall survival of three UCEC subtypes differed significantly. The hierarchical clustering analysis is capable of distinguishing patients with different prognosis. However, this method is complicated and produces irrelevant information, making it less clinically applicable.

To overcome the shortcomings, we filtered out two hub genes (CCL13 and KLRC1) closely related to the prognosis of UCEC patients. The mRNA and protein expression levels of both genes were verified via qPCR and IHC. The survival analyses confirmed the ability of IRPS in distinguishing patients with different prognosis. In order to enhance the predictive power of the IRPS, we added other clinical characteristics and built a nomogram model. According to the ROC and DCA curves, this nomogram model exhibited remarkable ability in predicting the prognosis of patients.

We further investigated the potential biological function of IRPS. The GSEA analysis revealed that several immune-related pathways were significantly enriched in the low-risk group. In contrast, the same pathways in the high-risk group were scattered. It is widely acknowledged that innate and adaptive immune cells play a major role in regulating the cancer growth. Increasing evidence shows that some immune cells (like Neutrophils, Macrophage M2, T regulatory cell) can stimulate, while some (like Macrophage M1, CD8+ T cell and Th1 CD4+ T cell) can inhibit cancer growth (Coussens and Werb, 2002; Disis, 2010). In the present study, low-risk and high-risk groups differed in the proportion of immune cells in UCEC, including plasma cells, CD8+ T cells, CD4+ memory T cells, CD4+ memory T cells, follicular helper T cells, M0 macrophages, M1 macrophages and activated mast cells. To be specific, M1 Macrophages, CD4+ and CD8+ T cells and plasma cells were activated in the low-risk group, suggesting that they can inhibit cancer growth and improve the prognosis of UCEC patients.

As listed in the methods section, the IRPS was established using the expression profiles of CCL13 and KLRC1. CCL13 is a gene located on chromosome 17q11.2 that encodes monocyte chemoattractant protein 4 (MCP-4), a Cys-Cys (CC) type cytokine characterized by two adjacent cysteines. In the immunoregulatory and inflammatory processes, CCL13 demonstrates chemotaxis to monocytes, lymphocytes, basophils and eosinophils, but not neutrophils, and plays a role in the accumulation of leukocytes during inflammation. Increasing evidence has confirmed that chemokines and their receptors can facilitate the entry of specific immune cells into tumors, thus enhancing anti-tumor response and improving patient prognosis (Rusakiewicz et al., 2013; Jacquelot et al., 2018).

KLRC1 (Killer Cell Lectin Like Receptor C1), also known as NKG2A, is a protein-coding gene associated with Natural killer (NK) cells. NK cells can mediate the lysis of certain tumor cells and virus-infected cells, and specific humoral and cell-mediated immunity. The protein encoded by KLRC1 belongs to the killer cell lectin-like receptor family, also called NKG2 family, which is a group of transmembrane proteins preferentially expressed in NK cells. KLRC1 can form a complex with KLRD1/CD94 and participate in the recognition of the MHC class I HLA-E molecules in NK cells. Researcher has also proved that crystal structure of CD94-NKG2A in complex with HLA-E bound to a peptide derived from the leader sequence of HLA-G. A previous study found KLRC1 expression changed with CD8+ T cell infiltration in 34 types of human cancers (Chen et al., 2019).

It is well known that tumors can escape the immune system via several mechanisms, including expanding T regulatory cells, inducing the production of certain inhibitory cytokines, altering the function of antigen presenting cells (APCs) (Disis, 2010). In this research, we found that the expression of CCL13 and KLRC1 had a positive correlation with the activation of several types of immune cells. Mutations in these two genes can inhibit the infiltration of some immune cells, especially in CD8+ T cells. Thus, IRPS based on both genes can distinguish cellular immunoactivation and immunosuppression.

We also investigated whether IRPS can provide valuable information about the host response to immunotherapy and chemotherapy. Immune checkpoint molecules are traditional biomarkers for evaluating the therapeutic benefit of immunotherapy. In this research, we found that the expression levels of four immune checkpoint molecules (PD-1, PD-L1, PD-L2 and CTLA4) were significantly low in the high-risk group, suggesting that the patients in the high-risk group might not benefit from immunotherapy based on immune checkpoint inhibitors. Apart from immune checkpoint molecules, tumor mutational burden (TMB) has emerged as a promising predictive biomarker for immunotherapy based on immune checkpoint inhibitors in several tumor types (Chen et al., 2019). High TMB and high neoantigen load have positive correlation with sensitivity to immunotherapy (Hollern et al., 2019). Multiple studies have proved that TMB may be a surrogate for overall neoantigen load^20^ (Rizvi et al., 2015; Rooney et al., 2015). During the cancer onset, somatic cells mutate and express neoantigens (Gubin et al., 2015). These neoantigens can sometimes induce T-cell-dependent immune responses by activating CD8+ T cells that can recognize those neoantigens and initiate tumor cell lysis (Chen and Flies, 2013).

In this research, when it turns to the evidence regarding immune checkpoint molecules, According to TMB and ImmuCellAI tool, patients in the low-risk group may benefit more from immunotherapy with immune checkpoint inhibitors, but those in the high-risk group may not. Similarly, the patients in the low-risk group were sensitive to chemodrugs such as cisplatin, gemcitabine, paclitaxel, bleomycin, vinblastine, vinorelbine, vorinostat and methotrexate. However, patients in the high-risk group were resistant to these drugs, which may explain their poor prognosis.

Fortunately, we found that several small molecule drugs, such as carbenoxolone, emetine, lovastatin and MG-262, could provide potential benefits for patients in the high-risk group. There is limited research regarding the effects of these drugs on tumor. Carbenoxolone is widely used as an antiulcer drug, but with unknown effect on tumor. Carbenoxolone can also act as the inhibitor of Pannexin 1 (Panx-1) and suppress the migration and invasion of testicular cancer cells to counter cancer progression and metastasis (Penuela et al., 2012; Furlow et al., 2015; Jankowski et al., 2018; Liu et al., 2019). Emetine, a potent anti-protozoal and emetic drug, recent evidence has verified its anti-malarial, anti-bacterial and anti-amoebic effects (Matthews et al., 2013; Hudson et al., 2016; Khandelwal et al., 2017; Yang et al., 2018). Over the past decades, emetine has been reported to have anti-tumor effects on leukemia, ovarian cancer, bladder cancer and lung cancer by inhibiting tumor growth by regulating multiple mechanisms such as apoptosis and autophagy (Moller and Wink, 2007; Moller et al., 2007; Kim et al., 2015; Sun et al., 2015). Lovastatin, an HMG-CoA reductase inhibitor, can decrease cholesterol biosynthesis and is an ideal medicine for treating coronary heart disease. In 2004, lovastatin was found to be a useful adjuvant drug for breast cancer (Shibata et al., 2004). Besides, lovastatin can reduce cancer-related deaths. MG-262, also known as Z-Leu-Leu-Leu-B(OH)2, is a proteasome inhibitor that can reversibly and selectively inhibit chymotryptic activity of the proteasome. As we know, proteasome inhibition has emerged as a novel approach to treat cancer. In some studies, MG-262 has exhibited obvious inhibitory effect on the growth of malignant cells.

The above drugs are untraditional anti-tumor drugs and there is limited evidence of their effects on tumors especially UCEC. However, for patients in the high-risk group, all drugs with potential benefits should be tried. For patients who may not benefit from traditional drugs, adjuvant agents should be tried.

Nevertheless, there are still some limitations in this research. First, this study only includes the immune-related genes and did not take other biomarkers into consideration. Another important issue, there are lots of immune cells varies significantly among individuals, it is hard to distinguish if the gene expression level mainly depends on the varieties of immune cells. The variable number of NK cells might be a confounder for KLRC1 expression. We used Timer2.0 to explore the correlation of KLRC1 and NK cells, the result confirmed that the expression of KLRC1 is positively correlated with NK cells. However, according to the results of CIBERSORT analysis, the proportion of NK cells showed no significant difference in high- and low- risk group. Besides, by analyzing the correlation between the RiskScore and different types of immune cells, we found the RiskScore was negatively correlated with NK cells. Thus, we speculated that the expression of KLRC1 might not mainly depend on the number of NK cells varieties among individuals. It was an independent prognosis related factor. Additionally, this research is based on the online data, and large-sample clinical studies are still needed to validate the predictive value of our IRPS model.

In summary, our study identified two immune-related genes, CCL13 and KLRC1 in the development of UCEC. The IRPS of both genes can predict the prognosis and immune status of UCEC patients and evaluate their therapeutic response to immunotherapy and chemotherapy.

## Data Availability Statement

The datasets presented in this study can be found in online repositories. The names of the repository/repositories and accession number(s) can be found in the article/Supplementary Material.

## Ethics Statement

The studies involving human participants were reviewed and approved by Clinical Research Ethics Committee, Wuxi Maternal and Child Health Hospital, The Affiliated Hospital to Nanjing Medical University. The patients/participants provided their written informed consent to participate in this study.

## Author Contributions

YZ conceived the study. JL, YW, and JM participated in the design, analysis, and draft of the study. JL and YW plotted all figures in this manuscript. JM helped in data analysis. All authors approved the final version of this manuscript and agreed to be accountable for all aspects of the work.

## Conflict of Interest

The authors declare that the research was conducted in the absence of any commercial or financial relationships that could be construed as a potential conflict of interest.

## Publisher’s Note

All claims expressed in this article are solely those of the authors and do not necessarily represent those of their affiliated organizations, or those of the publisher, the editors and the reviewers. Any product that may be evaluated in this article, or claim that may be made by its manufacturer, is not guaranteed or endorsed by the publisher.

## Footnotes

1 http://cancergenome.nih.gov/

2 https://www.immport.org

3 https://cistrome.shinyapps.io/timer

4 http://cis.hku.hk/TISIDB/index.php

5 https://portal.gdc.cancer.gov

6 https://tcia.at/home

7 http://gepia.cancer-pku.cn/index.html

8 https://www.cancerrxgene.org

9 https://www.broadinstitute.org/connectivity-map-cmap

10 https://pubchem.ncbi.nlm.nih.gov/

11 http://www.r-project.org/

## Abbreviations

UCEC: Uterine Corpus Endometrial Carcinoma
LASSO: least absolute shrinkage and selection operator
IRPS: immune-related prognostic signature
GEPIA: Gene Expression Profiling Interactive Analysis
GDSC: Genomics of Drug Sensitivity in Cancer
TMB: tumor mutational burden
TIME: tumor immune microenvironment
TCGA: the Cancer Genome Atlas
GO: gene ontology
KEGG: Kyoto Encyclopedia of Genes and Genomes
FDR: false discovery rate
ROC: receiver operating characteristic
AUC: area under the curve
DCA: decision curve analysis
GSEA: gene set enrichment analysis
TIICs: tumor-infiltrating immune cells
KLRC1: Killer Cell Lectin Like Receptor C1.
