# Supplementary material for: Identification of a Novel Immune Landscape Signature for Predicting Prognosis and Response of Endometrial Carcinoma to Immunotherapy and Chemotherapy
Source: Front Cell Dev Biol. 2021 Jul 23;9:671736. doi: 10.3389/fcell.2021.671736 (PMC8343236; doi:10.3389/fcell.2021.671736)
Supplement: Supplementary file 1 [file Data_Sheet_1.pdf]

## Supplementary data

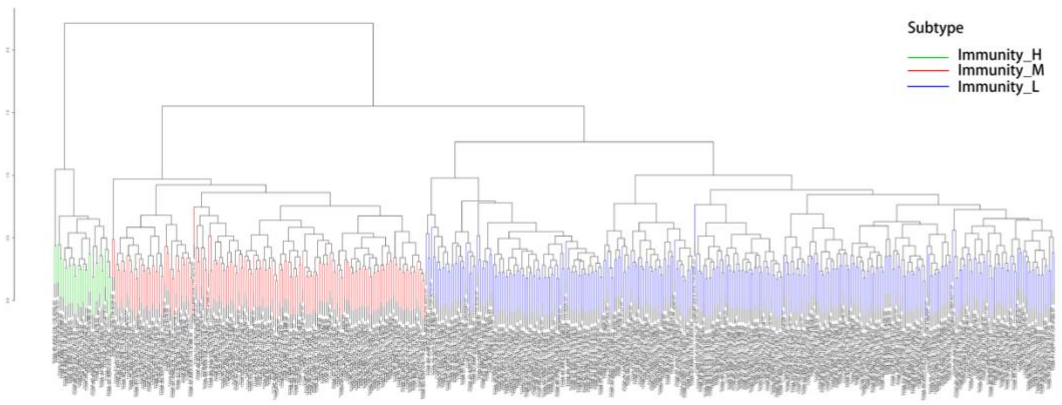


Figure S1. The hierarchical clustering map. Patients were divided into three clusters according to immune infiltration calculated by ssGSEA.

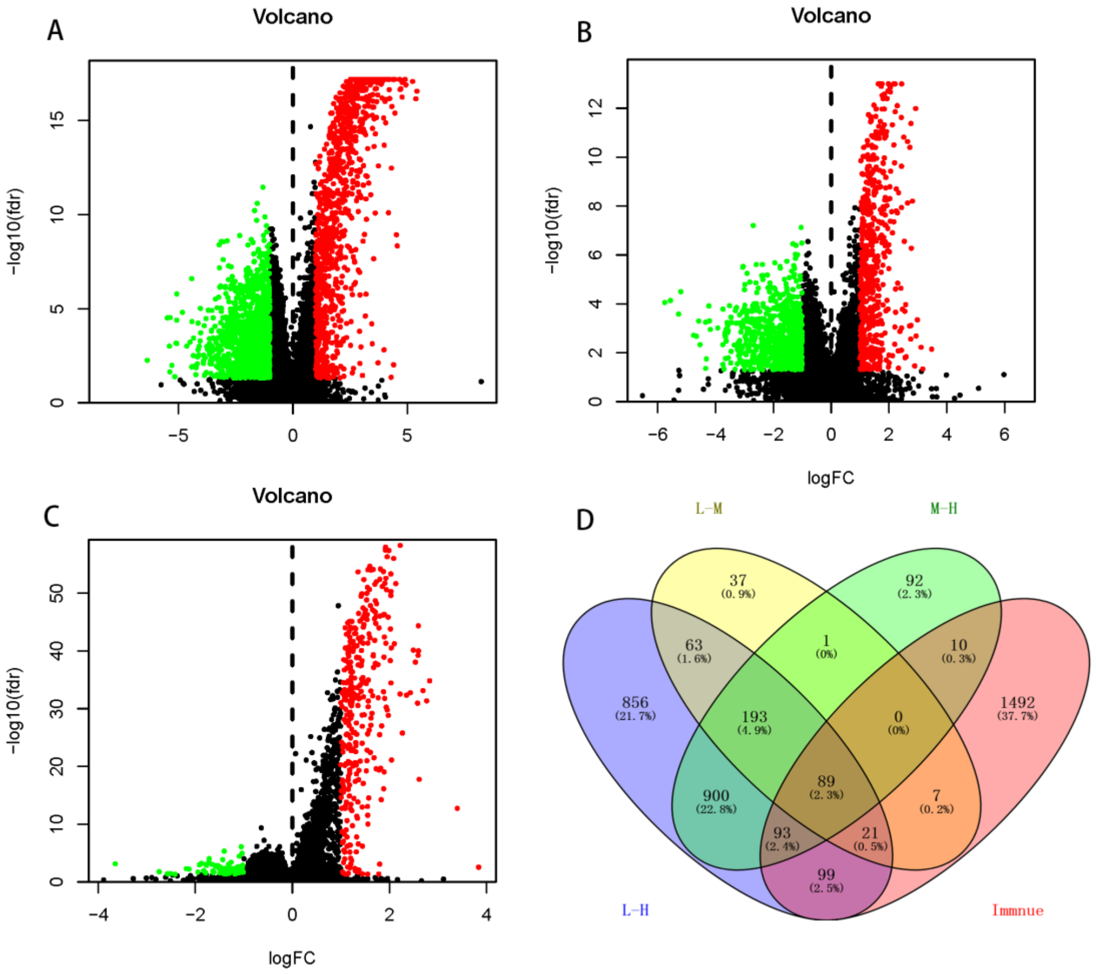


Figure S2. Differentially expressed genes (DEG) in three UCEC subtypes. (A-C) The

Volcano plots showed the DEGs between different groups (A: Immunity_L vs Immunity_H. B: Immunity_L vs Immunity_M. C: Immunity_M vs Immunity_H). (D) The Venn plot exhibited the intersections between DEGs and immune-related genes. Red points stand for up-regulated genes, while green point stand for down-regulated genes.

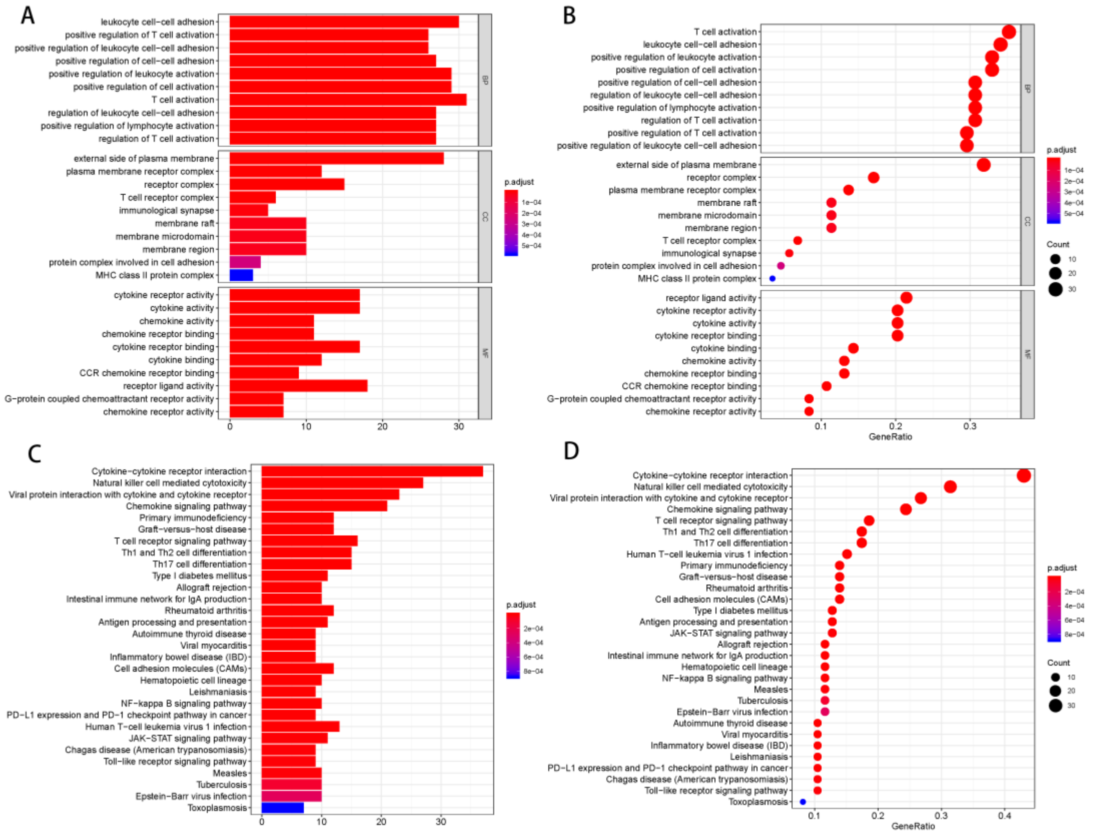


Figure S3. Functional enrichment analyses of DEGs. (A, B) Gene ontology (GO) analysis showed the enriched go term of these DEGs. (C, D) Kyoto Encyclopedia of Genes and Genomes (KEGG) analysis exhibited the enriched biological pathways of these DEGs.

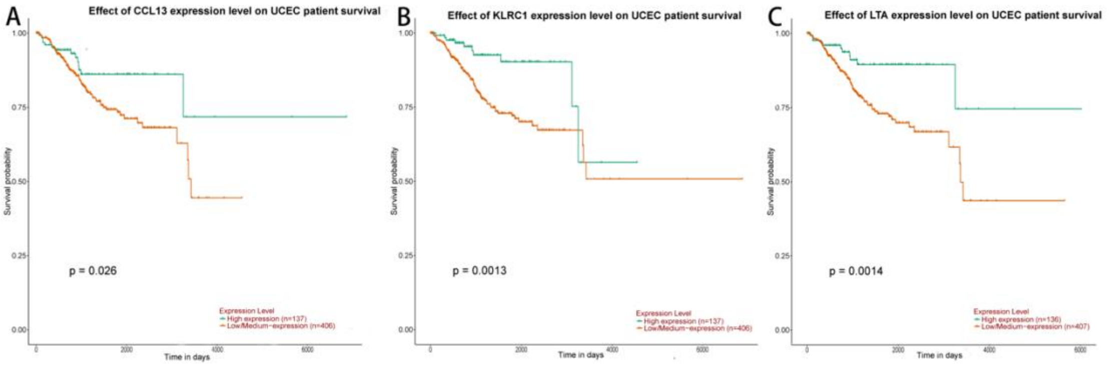


Figure S4. The effect of expression level of 3 target genes on overall survival of UCEC patients. (A) Effect of CCL13 expression level on UCEC patient survival. (B) Effect of KLRC1 expression level on UCEC patient survival. (C) Effect of LTA expression level on UCEC patient survival.

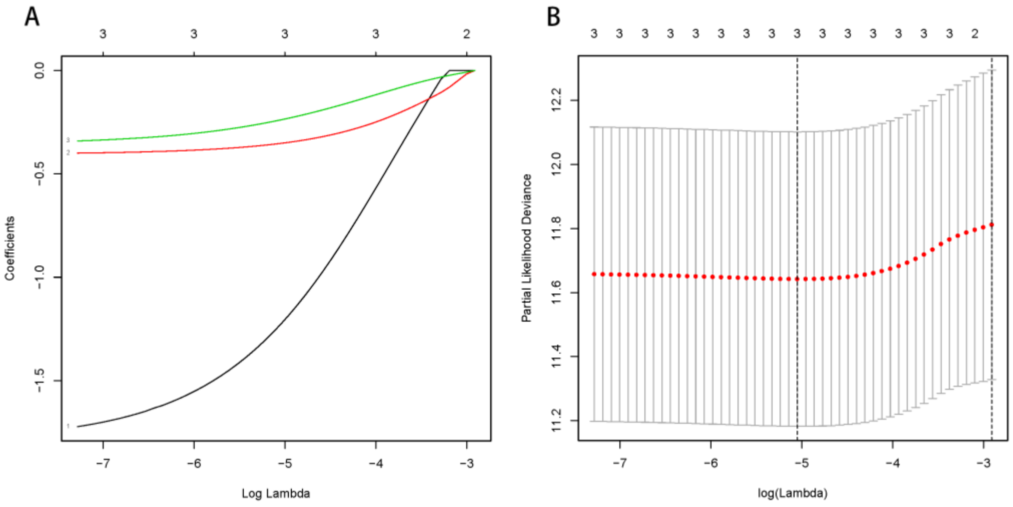


Figure S5. Lasso Cox regression analysis of 3 hub genes. (A) Lasso coefficient profiles of 3 hub genes. (B) 10-fold cross-validations results which identified optimal values of

the penalty parameter λ.

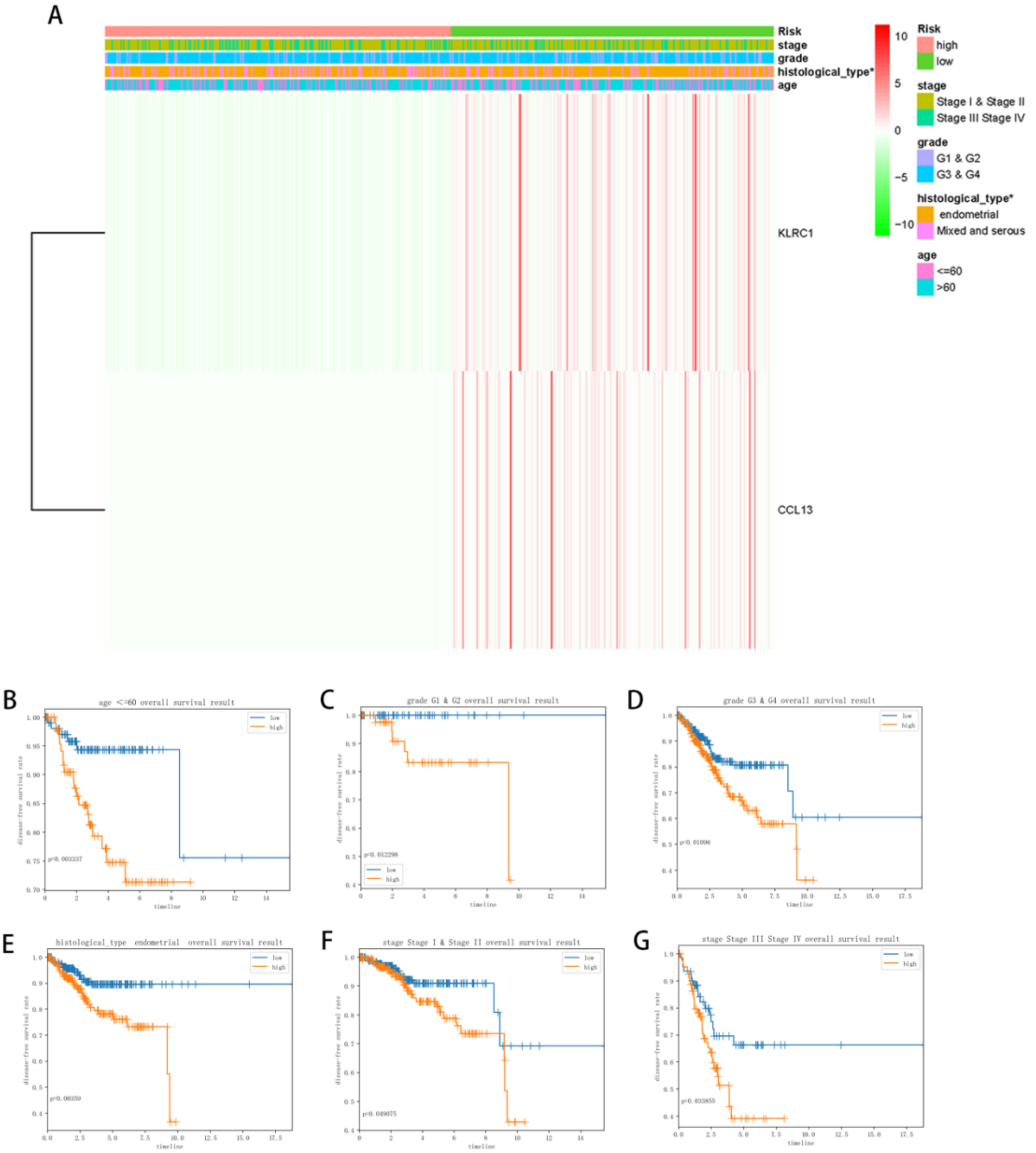


Figure S6. The prognostic impact of the IRPS under different clinical features. (A) The heatmap shows the distribution relationship of risk score, hub genes and other clinical features. (B-G) The survival analyses exhibited that IRPS reached satisfactory prognostic discrimination in patients with age ≤ 60, grade G1&G2, grade G3&G4, histological type endometrial, stage I&II and stage III&IV.

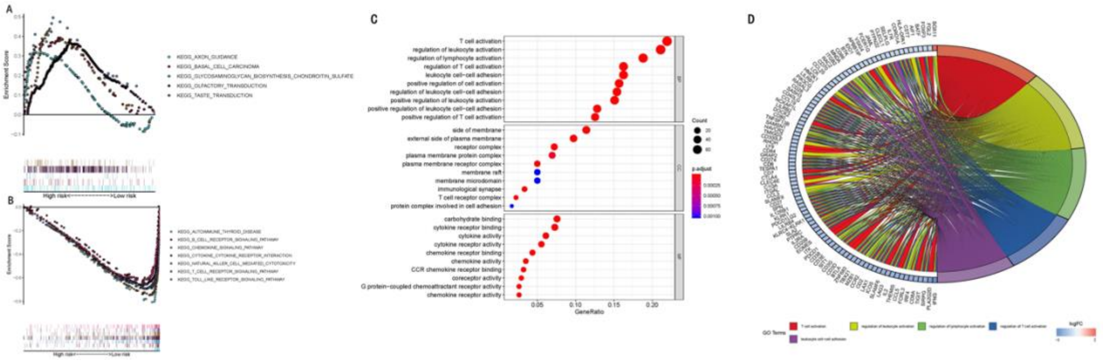

Figure S7. GESA and GO analysis of UCEC based on IRPS. (A) The enriched gene sets in high-risk group. (B) The enriched gene sets in low risk. (C) The GO enrichment of the DEGs. (D) The enrichment genes of the core GO terms.

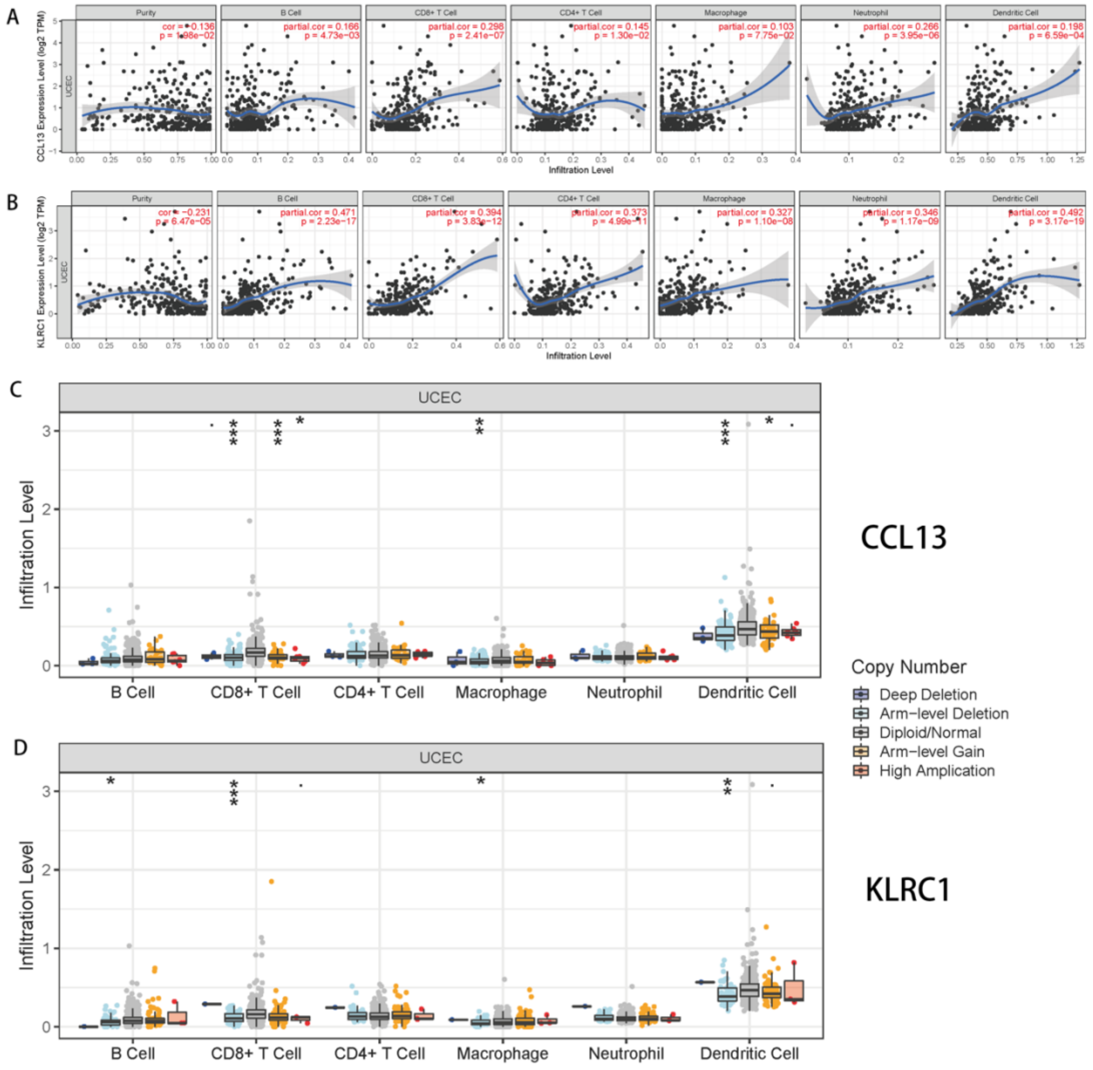


Figure S8. Correlation of 2 hub genes with tumor purity and immune filtrating cells.

(A) CCL13 expression is inversely related to tumor purity and has significant positive correlations with infiltrating levels of B cell, CD8+ T cell, CD4+ T cell, neutrophils, and dendritic cells in UCEC, other than macrophages. (B) KLRC1 expression is also significantly negatively related to tumor purity and has positive correlation with infiltrating levels of B cells, CD8+ T cells, CD4+ T cells, macrophages, neutrophils, and dendritic cells in UCEC. (C) Mutants of CCL13 were associated with low infiltration of CD8+ T cell, macrophage and dendritic cell. (D) Mutants of KLRC1 were associated with low infiltration of B cell, CD8+ T cell, macrophage and dendritic cells.

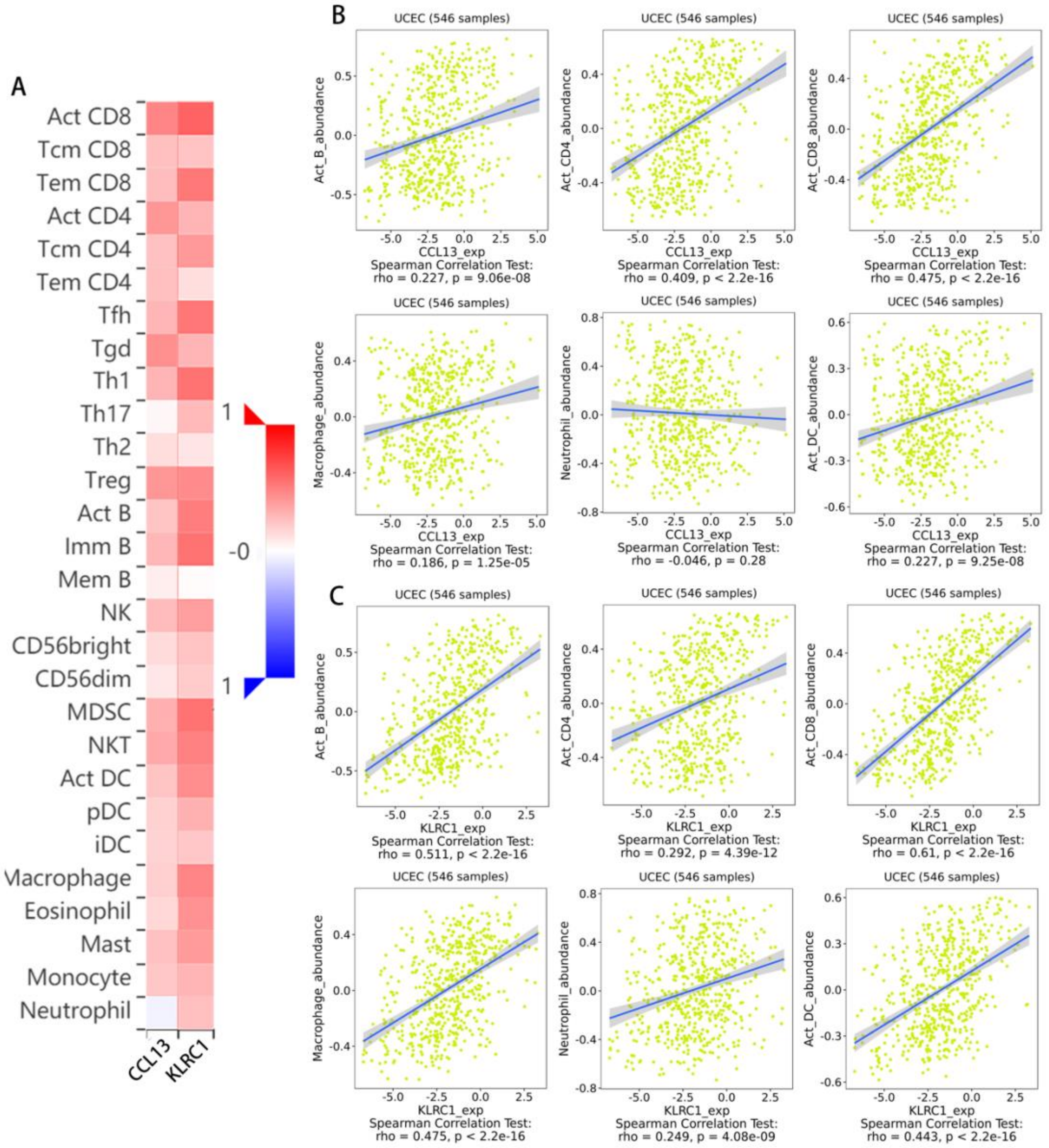


Figure S9. Correlation between the expression of 2 hub genes and immune filtrating cells in UCEC. (A) Correlation between the expression of 2 hub genes and the abundance of immune filtrating cells in UCEC available at TISIDB database. (B) Correlation of CCL13 expression with infiltration levels of B cell, CD4+ T cell, CD8+ T cell, macrophages, neutrophils and dendritic cells in UCEC. (C) Correlation of KLRC1 expression with infiltration levels of B cells, CD4+ T cells, CD8+ T cells, macrophages, neutrophils and dendritic cells in UCEC.

Table S1. Univariate cox analysis of 89 differentially expressed genes

| Gene | HR | 95%CI | p value |
|---|---|---|---|
| CCL13 | 0.638 | 0.4110-0.994 | 0.047 |
| LTA | 0.332 | 0.111-0.988 | 0.047 |
| KLRC1 | 0.071 | 0.005-0.994 | 0.050 |

Table S2. Primers used in PCR application

| Gene | Forward primers | Reverse primer |
|---|---|---|
| CCL13 | CTCAACGTCCCATCTACTTGC | TCTTCAGGGTGTGAGCTTTCC |
| KLRC1 | AGCTCCATTTTAGCAACTGAACA | CAACTATCGTTACCACAGAGGC |
| GAPDH | ACCACAGTCCATGCCATCAC | TCTAGACGGCAGGTCAGGTC |
