# Supplementary material for: Identification of a Novel Immune Landscape Signature for Predicting Prognosis and Response of Endometrial Carcinoma to Immunotherapy and Chemotherapy
Source: Front Cell Dev Biol. 2021 Jul 23;9:671736. doi: 10.3389/fcell.2021.671736 (PMC8343236; doi:10.3389/fcell.2021.671736)
Supplement: Supplementary file 3 [file Table_1.DOCX]

Table S1. Univariate cox analysis of 89 differentially expressed genes

| Gene | HR | 95%CI | p value |
| --- | --- | --- | --- |
| CCL13 | 0.638 | 0.4110-0.994 | 0.047 |
| LTA | 0.332 | 0.111-0.988 | 0.047 |
| KLRC1 | 0.071 | 0.005-0.994 | 0.050 |
