# Supplementary material for: Identification of a Novel Immune Landscape Signature for Predicting Prognosis and Response of Endometrial Carcinoma to Immunotherapy and Chemotherapy
Source: Front Cell Dev Biol. 2021 Jul 23;9:671736. doi: 10.3389/fcell.2021.671736 (PMC8343236; doi:10.3389/fcell.2021.671736)
Supplement: Supplementary file 4 [file Table_2.DOC]

Table S2. Primers used in PCR application

| Gene | Forward primers | Reverse primer |
| --- | --- | --- |
| CCL13 | CTCAACGTCCCATCTACTTGC | TCTTCAGGGTGTGAGCTTTCC |
| KLRC1 | AGCTCCATTTTAGCAACTGAACA | CAACTATCGTTACCACAGAGGC |
| GAPDH | ACCACAGTCCATGCCATCAC | TCTAGACGGCAGGTCAGGTC |
